# Nanosphere Lithography-Enabled Hybrid Ag-Cu Surface-Enhanced Raman Spectroscopy Substrates with Enhanced Absorption of Excitation Light

**DOI:** 10.3390/bios13080825

**Published:** 2023-08-17

**Authors:** Zixuan Wu, Jianxun Liu, Zhenming Wang, Lei Chen, Yiwei Xu, Zongjun Ma, Delai Kong, Dan Luo, Yan Jun Liu

**Affiliations:** 1 Department of Electrical and Electronic Engineering, Southern University of Science and Technology, Shenzhen 518055, China; 12012007@mail.sustech.edu.cn (Z.W.); 12150025@mail.sustech.edu.cn (Z.W.); 12132106@mail.sustech.edu.cn (L.C.); 12011843@mail.sustech.edu.cn (Y.X.); 12031132@mail.sustech.edu.cn (Z.M.); 12232158@mail.sustech.edu.cn (D.K.); luod@sustech.edu.cn (D.L.); 2 Shenzhen Engineering Research Center for High Resolution Light Field Display and Technology, Southern University of Science and Technology, Shenzhen 518055, China

**Keywords:** SERS, enhancement factor, hot spot, self-assembly, nanosphere lithography

## Abstract

We demonstrated a low-cost, highly sensitive hybrid Ag-Cu substrate with enhanced absorption for the excitation laser beam via the nanosphere lithography technique. The hybrid Ag-Cu surface-enhanced Raman spectroscopy (SERS) substrate consists of a Cu nanoarray covered with Ag nanoparticles. The geometry of the deposited Cu nanoarray is precisely determined through a self-assembly nanosphere etching process, resulting in optimized absorption for the excitation laser beam. Further Raman enhancement is achieved by incorporating plasmonic hotspots formed by dense Ag nanoparticles, grown by immersing the prepared Cu nanoarray in a silver nitrate solution. The structural design enables analytical enhancement factor of hybrid Ag-Cu SERS substrates of 1.13 × 10^5^. The Ag-Cu SERS substrates exhibit a highly sensitive and reproducible SERS activity, with a low detection limit of 10^−13^ M for Rhodamine 6G detection and 10^−9^ M for 4,4′-Bipyridine. Our strategy could pave an effective and promising approach for SERS-based rapid detection in biosensors, environmental monitoring and food safety.

## 1. Introduction

Surface-enhanced Raman spectroscopy (SERS) has emerged as a powerful analytical technique for sensitive molecule detection and characterization [1,2,3]. The label-free, non-destructive and ultrasensitive characteristics [4,5] of SERS have led to wide applications in diverse fields, including monitoring toxic pollutants [6], biomedical research [7] and food safety [8]. In SERS, the enhancement of Raman signals can be achieved through two main physical mechanisms: the electromagnetic enhancement and the chemical enhancement mechanism [9,10,11,12,13,14]. Electromagnetic enhancement can be attained through both non-plasmonic and plasmonic substrates. Various studies for SERS based on non-plasmonic substrates have been proposed, such as Si@SiO_2_ Q-probes [15], ZnO-based 3D semiconductor quantum probes [16], and MoO_3_·xH_2_O quantum dots [17]. Compared to non-plasmonic substrates, the SERS effect based on electromagnetic enhancement is more effectively achieved using densely distributed plasmonic nanostructures of Au, Ag, and Cu on a substrate, creating numerous “hot spots” [18,19,20,21]. These nanostructures rely on the localized surface plasmon resonance (LSPR) effect, resulting from the collective oscillation of conduction electrons upon light excitation, thereby generating a strong electromagnetic field enhancement near the metal surface [1,19]. In particular, various Ag or hybrid Ag-Cu nanostructure-based SERS substrates have been heavily investigated [22,23,24]. However, the Cu nanostructures serving as the growth base have been rarely utilized to enhance the electromagnetic field in previous reports, presenting an opportunity to involved Cu in electric field enhancement through a cost-effective approach.

It has been reported that the absorption of excitation light in Raman experiments can effectively improve the electromagnetic field enhancement [25,26,27]. Various nanostructures have been developed to enhance the absorption of incident laser [28,29,30]. The enhancement of incident laser absorption amplifies the optical density within a defined space, thereby intensifying the interactions between photons and absorbed molecules [31,32,33]. The high optical density would form a strong focusing field [33], which amplifies the Raman scattering signal from absorbed molecules. With the aid of advanced fabrication techniques, such as electron beam lithography (EBL) and focused ion beam (FIB), it is possible to achieve precisely controlled metal nanoarrays with resonant absorption properties. However, these precise nanofabrication techniques involve high-cost and complicated fabrication processes. A facile and cost-effective technique for nanoarrays fabrication is therefore highly demanded. With hexagonal close-packed or face-centered cubic lattices, self-assembly-based nanosphere lithography (NSL) [34,35,36] is a low-cost, large-area, and simple fabrication technique that has aroused intensive attention. NSL utilizes the self-assembled monolayer of nanospheres as a template mask to produce uniform and symmetrical periodic structures. Using the small size nanospheres in NSL, one can achieve characteristic wavelength absorption properties similar to those obtained through EBL and FIB techniques [37,38,39]. Various periodic nanostructures based on a polystyrene (PS) monolayer mask, such as nanosteps [40], nanohole arrays [41], and nanotriangle arrays [42], have been utilized to significantly enhance Raman signals. In our previous work, we successfully prepared SERS substrates with strong electromagnetic field enhancement at multiple wavelengths via NSL [40,43,44].

In this work, we present the fabrication of a hybrid Ag-Cu substrate based on the NSL technique, incorporating a Cu nanoarray design and subsequent growth of Ag nanoparticles via replacement reaction. By precisely controlling the time of the inductively coupled plasma (ICP) etching process, we optimize the structure of Cu nanoarrays to enhance the absorption at the excitation laser wavelength of 532 nm. Furthermore, Ag nanoparticles are grown on fabricated Cu nanoarrays, and the morphologies of Ag nanoparticles are controlled by varying the reaction time. The Cu nanoarrays and Ag nanoparticles have a collective effect for the electromagnetic enhancement of the Raman signal. As a result, the fabricated hybrid Ag-Cu SERS substrates demonstrate an analytical enhancement factor (AEF), exhibiting excellent SERS performance in trace molecule detection. Our proposed method could pave the way for an effective and promising approach for SERS-based rapid detection in biosensors, environmental monitoring and food safety.

## 2. Materials and Methods

### 2.1. Materials

PS nanosphere water solutions with a concentration of 2.5 wt.% were procured from Alfa Aesar (China) Chemical Co., Ltd. (Shanghai, China). The PS nanospheres have a uniform size of 500 nm in diameter. Standard solutions required for silver nitrate titration analysis with a concentration of 0.1010 m/L, were obtained from Beijing Merida Technology Co., Ltd. (Beijing, China). Rhodamine 6G (R6G, C_28_H_31_N_2_O_3_Cl) and 4,4′-Bipyridine (BPy, C_10_H_8_N_2_, purity ≥ 98%) were purchased from Sigma-Aldrich (Shanghai, China) Trading Co., Ltd. (Shanghai, China), and Shanghai Aladdin Bio-Chem Technology Co., Ltd. (Shanghai, China), respectively. Silicon wafers with a size of 150 mm in diameter (P-Type, <100>) were procured from Beijing Youran Yisen Technology Co., Ltd. (Beijing, China). Glass (calcium sodium glass) was purchased from Luoyang Tengchang Xukun Biotechnology Co., Ltd. (Luoyang, China).

### 2.2. SERS Substrates Preparation

The Si and glass substrates were meticulously cut into 2 cm × 2 cm pieces and subjected to a 20 min treatment with a UV-O_3_ cleaner (BZS250GF-TC, HWOTECH, Shenzhen, China). Using the split injection pump (RSP01-BD, BIOTAOR, Jiaxing, China), high-quality templated PS monolayer masks were prepared via the air–liquid interface method. The following are the detailed PS monolayer self-assembly processes. First, the Si substrate was placed on a hot plate with the temperature set as 45 °C, and then an appropriate amount of deionized (DI) water was added onto the substrate surface. Subsequently, a mixed PS solution (the volume ratio of 2.5 wt.% PS aqueous solution and ethanol is 2:1) was applied to a designated corner of the substrate using the split injection pump with precise control. Given the hydrophobic characteristics of the PS nanospheres, the DI water on the Si substrate surface was forced to diagonal region. However, over time, the DI water gradually recovered the substrate area. Therefore, the above process can only be realized by adding the appropriate amount of DI water to the Si substrate. Following the self-assembly process, the templated PS monolayer masks were transferred onto a glass substrate using the method described in our previous reports [40,43]. After the successful transfer of the templated mask onto glass substrates, the inductively coupled plasma (ICP) (GSE200Plus, Northern Microelectronics, Gateshead, UK) etching technique was employed to etch the PS nanospheres using O_2_ plasma. The etching process was conducted at a pressure of 8 mTorr, an O_2_ flow of 50 sccm, an ICP power of 100 W and a bias power of 20 W. Etching durations of 60, 80, 120, 140 and 160 s were applied for the PS nanospheres. Following the etching process, a 30 nm Cu layer was deposited onto the etched templated mask using an electron beam evaporator (TF500, British HHV, Crawley, UK). Following the deposition process, the Cu-coated templated masks were subjected to immersion in deionized water, facilitating the ultrasonic removal of PS nanospheres (ultrasonic time: 5 min). It is worth noting that the surface of the deposited Cu layer is susceptible to oxidation, necessitating a de-oxidation step for the Cu nanoarrays before the galvanic replacement reaction can take place. This de-oxidation process ensures the removal of surface oxides, thus providing fresh and clean Cu surfaces for the subsequent electrochemical reaction, enabling optimal performance of the hybrid Ag-Cu SERS substrates. To clean the Cu surfaces, the Cu nanoarray substrates were immersed in a 1% diluted sulfuric acid solution (3 min), effectively eliminating surface oxides. Afterwards, thorough rinsing with DI water was carried out to ensure the removal of any residual acid and contaminants, resulting in clean and pristine Cu surfaces that were ready for further processing and experimentation. Subsequently, the pretreated Cu nanoarray substrates were immersed in an aqueous solution of AgNO_3_ with a concentration of 3.33 mM, enabling the deposition of Ag nanoparticles on the Cu nanoarray substrates through the galvanic replacement reaction. Finally, the resulting hybrid Ag-Cu substrates were rinsed with DI water, rendering them ready for SERS tests.

### 2.3. Characterization and Measurement

The surface morphologies of the Cu nanoarrays and the hybrid Ag-Cu substrates were characterized using a field-emission scanning electron microscopy (FESEM, Merlin, Zeiss, Jena, Germany). The optical properties of the Cu nanoarray substrates were meticulously recorded using a UV-Vis-NIR microspectrophotometer (CRAIC Technologies Inc., Altadena, CA, USA). To evaluate the SERS performance, a confocal Raman system (Alpha300, WITec, Ulm, Germany) was employed. In the confocal Raman system, the diffraction gratings have a groove density of 1800 g/mm, and the blaze wavelength of the diffraction gratings is 500 nm. A laser beam (operating wavelength: 532 nm) was focused onto the samples using an objective lens (50×, numerical aperture (NA) = 0.5), and the Raman signal was collected. The excitation laser was set at a power of 100 μW/μm^2^, and each spectrum was acquired over a period of 5 s. We conducted a Raman mapping with a size of 5 × 5 μm^2^ and randomly selected 18 points to collect their SERS spectra. For the Raman mapping, the excitation laser was set at a power of 100 μW/μm^2^, and the period of collection for each spectrum was 2 s.

## 3. Results and Discussion

### 3.1. Fabrication and Characterization of Hybrid Ag-Cu SERS Substrate

**Figure 1:**
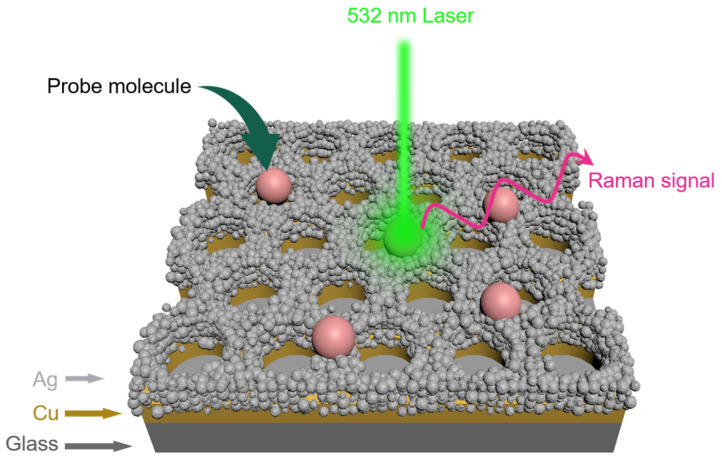
Schematic representation of trace molecule measurement using the hybrid Ag-Cu surface-enhanced Raman spectroscopy (SERS) substrate. The pink spheres represent the probe molecules.

Figure 1 illustrates the schematic representation of trace molecule measurement using the hybrid Ag-Cu SERS substrate. The substrate comprises a 1.1 mm thick glass layer, a 30 nm thick Cu nanoarray layer, and, finally, a coating of Ag nanoparticles achieved through the galvanic replacement reaction, arranged sequentially from the bottom to the top. The pink spheres depicted in Figure 1 represent the probe molecules, namely, R6G and BPy, utilized in our experiments. Notably, significant peaks on the SERS spectra are distinctly observed under the 532 nm laser excitation, which will be further elaborated in subsequent discussions.

**Figure 2:**
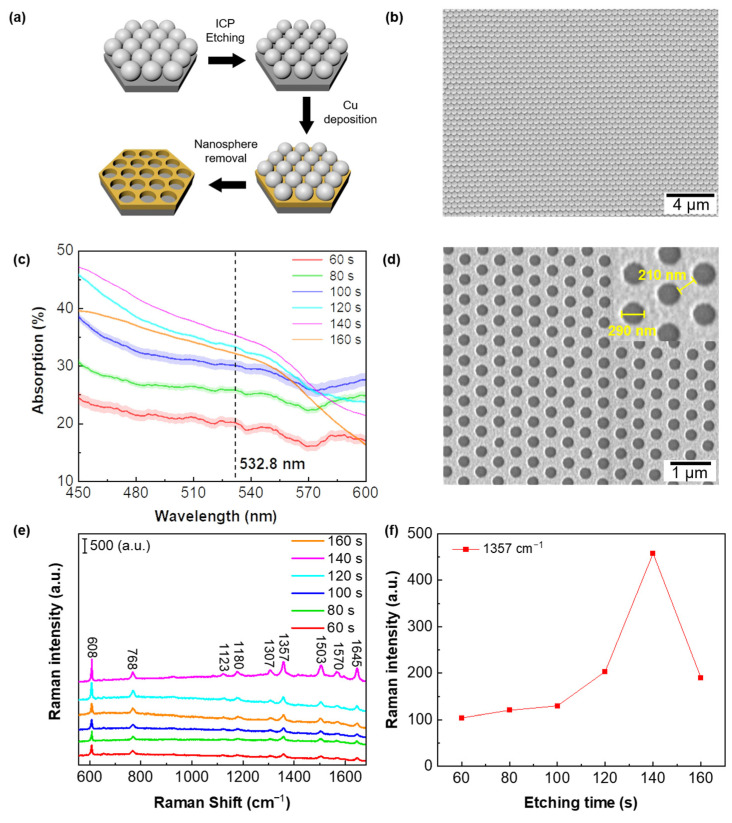
(**a**) The fabrication process for the Cu nanoarray. (**b**) Scanning electron microscopy (SEM) image displaying the high-quality templated PS monolayer mask. (**c**) Absorption spectra of the Cu nanoarrays featuring various sizes of nanoholes. (**d**) SEM images showcasing the Cu nanoarray with an etching time of 140 s. (**e**) SERS spectra obtained for 10^−6^ M R6G on hybrid Ag-Cu substrates (Ag nanoparticle growth time: 7 min) resulting from different etching times, namely 60, 80, 100, 120, 140 and 160 s. (**f**) Raman intensity of R6G at 1357 cm^−1^ plotted against the etching time as a parameter.

Figure 2a presents the schematic illustration of the fabrication processes involved in creating the Cu nanoarray, involving ICP etching, Cu deposition, and nanosphere removal. The high-quality templated PS monolayer mask is depicted in Figure 2b. From the SEM image, it is evident that the PS monolayer exhibits a uniform crystalline phase and shows no point defects within the hundreds of square micron range. High-quality PS monolayer masks play a pivotal role in nanofabrication, and their high-precision preparation process holds the potential to significantly advance the development of nanomaterials and devices. In general, low surface tension facilitates the formation of a film on the liquid surface, increasing the likelihood of nanospheres coming into close proximity and aggregating. However, excessively low surface tension would compromise the stability of the self-assembled structure. Therefore, achieving the proper surface tension of water is crucial in preparing high-quality PS monolayer masks [43,45]. In our experiments, we rigorously regulate the temperature to ensure precise control of the surface tension. In our experimental investigations, we employed the split injection pump, a technique known for controlling the quality of PS monolayer masks [46]. The parameters of this pump device were meticulously controlled to ensure optimal self-assembly process. Moreover, we exercised rigorous control over the substrate cleaning procedure and the ethanol to PS aqueous solution ratio. These comprehensive controls over experimental parameters ensure the reproducibility of high-quality PS monolayer masks. Based on high-quality PS monolayer masks, large-area Cu nanoarrays can be prepared via NSL technique. The diameter of the nanospheres decreases with increased ICP etching time. Indeed, the diameter of the nanosphere used in the templated PS monolayer mask determines the size of the nanoholes in the resulting Cu nanoarray. Hence, the etching time during the fabrication process plays a crucial role in optimizing the diameter of these nanoholes within the Cu nanoarrays. The primary objective of this optimization is to enhance both the excitation laser absorption and the resulting Raman signals. By carefully controlling the etching time, we aim to achieve Cu nanoarrays with the most efficient absorption of the excitation laser. This optimization process will contribute to maximizing the enhancement of Raman signals, ultimately leading to improved SERS performance for trace molecule measurements. Figure 2c shows the absorption spectra with different etching times. To ensure scientific validity and robustness of the experimental data, we conducted five separate measurements of the absorption spectra for each substrate with different etching times in Figure 2c. These measurements were performed at five random locations on each substrate within a 50 × 50 μm^2^ area. Subsequently, the collected data underwent rigorous processing and analysis to generate error band plots. We selected various positions on the Cu nanoarrays for spectral testing and generated error band diagrams. These error band diagrams help us to understand the homogeneity and reproducibility of the Cu nanoarrays. The error band diagrams reveal that the Cu nanoarrays exhibit excellent homogeneity and reproducibility. This finding underscores the substrate’s high-quality fabrication. As the etching time increases, there is a noticeable reduction in the diameter of the nanospheres, resulting in a decrease in the size of the nanoholes while simultaneously increasing the gap between them. Consequently, Cu nanoarrays with different etching times exhibit distinct light absorption at the wavelength of 532 nm. In our experiments, the fabricated Cu nanoarray with the etching time of 140 s has the strongest absorption. Figure 2d displays the SEM image of the corresponding Cu nanoarray with an etching time of 140 s. As evident from the SEM image, the Cu nanoarrays fabricated through the NSL technique exhibit a highly favorable periodic structure. The nanoholes in the Cu nanoarray have a diameter of 290 nm, and the gap between nanoholes measures 210 nm. In order to verify the absorption enhancement of Cu nanoarrays, a subsequent step was the growth of Ag nanoparticles on the corresponding Cu nanoarrays. This growth process was achieved by immersing the Cu nanoarrays in a 3.33 mM AgNO_3_ solution for a duration of 7 min. Their Raman spectra were measured for all the fabricated hybrid Ag-Cu substrates. We determined the reaction time to be 7 min through pre-experiments, and these data are presented in Figure S1 in the Supplementary Materials. Figure 2e illustrates the measured Raman spectra obtained for 10^−6^ M R6G on different Ag-Cu substrates resulting from varied etching times of 60, 80, 100, 120, 140 and 160 s, respectively. The intensity of the Raman signal shows an initial increase and subsequent decrease with increasing etching time. This observation indicates that there is an optimal etching time that maximizes the Raman signal enhancement on the Cu nanoarray substrate. Among these substrates, the Ag-Cu substrate with a 140 s etching time exhibits the strongest Raman signal enhancement. In most studies, the Raman characteristic peaks of R6G are primarily focused at 608 cm^−1^ [25], 1200 cm^−1^ [47], 1357 cm^−1^ [48] and 1509 cm^−1^ [49]. In our study, we observed strong enhancement at 1357 cm^−1^ compared to other characteristic peaks. Therefore, we chose to focus on the characteristic peak at 1357 cm^−1^. Figure 2f presents the Raman intensity of R6G at 1357 cm^−1^ (corresponding to the C–C stretching mode of R6G) plotted as a function of the etching time. From Figure 2f, it clearly shows that the Raman signal intensity varies with the etching time, confirming the effect of absorption enhancement by the Cu nanoarray. It indicates that the etching process plays a crucial role in optimizing the substrate’s light absorption capability. Comparing the absorption and Raman spectra reveals a positive correlation, providing further evidence of the absorption enhancement effect facilitated by the Cu nanoarray. Notably, the observed Raman peaks at 1123, 1180, 1307, 1357, 1503, 1570 and 1645 cm^−1^ are related to the vibrational modes of R6G molecules, demonstrating good agreement with previous reports [50,51,52].

**Figure 3:**
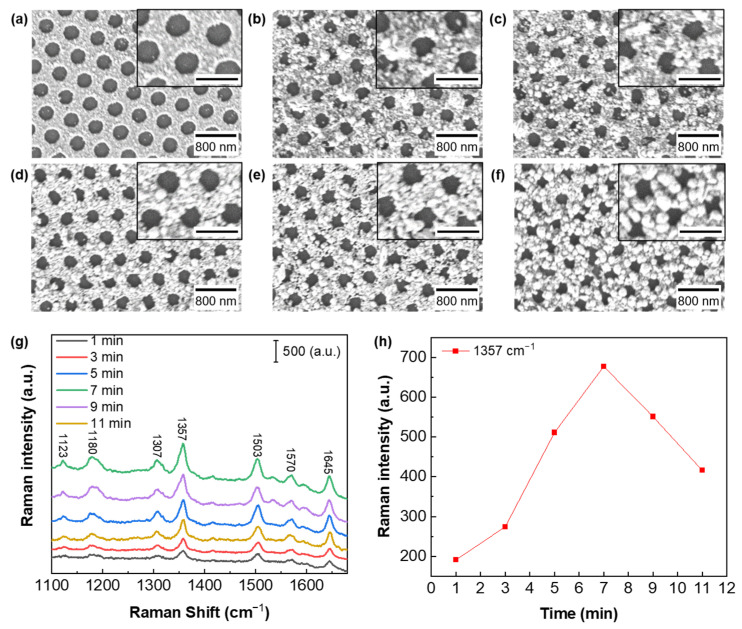
(**a**–**f**) SEM images of the hybrid Ag-Cu substrates acquired at different reaction times of 1, 3, 5, 7, 9 and 11 min, respectively. Insets show magnified images to illustrate clear morphologies with the scale bar of 500 nm. (**g**) SERS spectra obtained for 10^−6^ M R6G on different Ag-Cu substrates corresponding to reaction times of 1, 3, 5, 7, 9 and 11 min, respectively. (**h**) Raman intensity of R6G at 1357 cm^−1^ plotted as a function of the reaction time.

Upon achieving the optimized Cu nanoarray with the strongest absorption of excitation laser light, we conducted further optimization of the growth time for Ag nanoparticles on the Cu nanoarrays. The size of the Ag nanoparticles can be clearly resolved from Figure 3a–f. The obtained SEM images show the structural characteristics and morphological changes in the substrates during the reaction process. The size and density of Ag nanoparticles increase with prolonged reaction time and significantly impact the Raman enhancement [53,54,55,56]. For each substrate, we conducted a random selection of 100 Ag nanoparticles within an area of 5 × 5 μm^2^ and meticulously measured their diameters and distances between adjacent nanoparticles. The calculated average, variance, and standard deviation for all the statistical data are shown in Figures S2 and S3 in the Supplementary Materials. Figure S2 shows the size distributions of the Ag nanoparticles for different reaction times, while their distance distributions are presented in Figure S3. The statistics of the nanoparticle size and distance distributions are based on the SEM images in Figure 3a–f. Figure 3g presents the measured Raman spectra for 10^−6^ M R6G on hybrid Ag-Cu substrates obtained with reaction times ranging from 1 to 11 min. Additionally, Figure 3h describes the Raman intensity of R6G at 1357 cm^−1^ plotted as a function of the reaction time. Upon analysis of Figure 3g,h, we observe that the hybrid Ag-Cu substrate demonstrates the most robust Raman enhancement with the reaction time of 7 min. As the reaction time increases, the Raman enhancement initially increases and subsequently decreases. This observation is consistent with the widely recognized fact that the SERS effect strongly depends on the generation of “hot spots” created by noble metallic nanostructures [57,58]. When the reaction time for Ag nanoparticle growth is short, the resulting Ag nanoparticles tend to be small and sparse. This leads to a significant distance between neighboring nanoparticles, making it difficult for them to create substantial “hot spots” on the hybrid Ag-Cu substrate. When the reaction time is too long, Ag nanoparticles grow large and dense. The LSPRs of Ag nanoparticles might have a mismatch with the excitation wavelength, resulting in weak enhancement the SERS signal as well. Hence, there exists an optimal reaction time during which Ag nanoparticles are grown to provide the greatest number of closely spaced “hot spots” at the specific excitation wavelength. This optimal condition maximizes the SERS signal enhancement, facilitating highly sensitive and effective trace molecule detection and analysis. The selection of the optimal reaction time is crucial for achieving the best performance of the hybrid Ag-Cu substrate in SERS applications [22,59].

**Figure 4:**
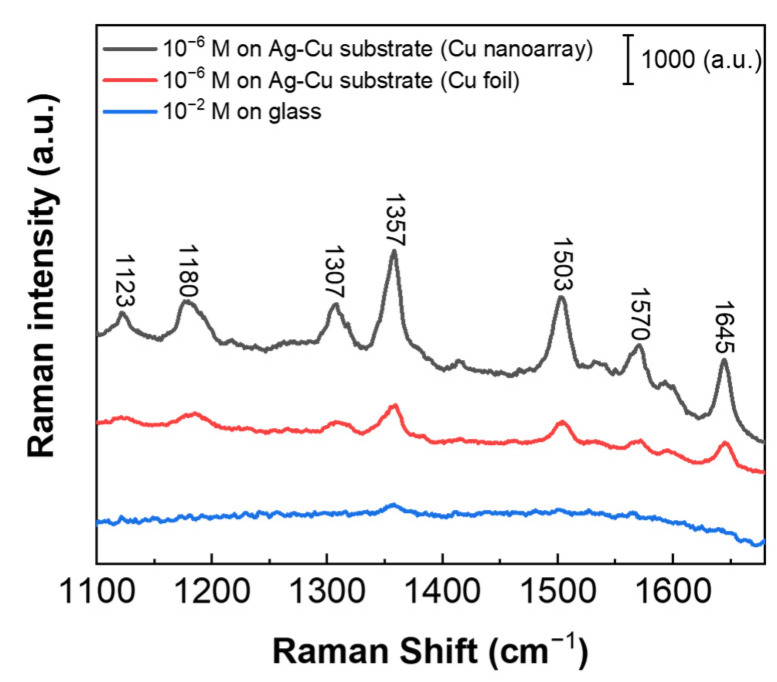
SERS spectra for the 10^−6^ M R6G on the hybrid Ag-Cu substrates with different Cu morphologies and 10^−2^ M R6G on the glass, respectively.

To quantitatively assess the enhancement distribution of the hybrid Ag-Cu SERS substrates, the analytical enhancement factor (*AEF*) is computed using the following equation [60]:

(1) $$AEF=\left(I_{\mathrm{SERS}}\times C_{\mathrm{REF}}\right)/\left(I_{\mathrm{REF}}\times C_{\mathrm{SERS}}\right)$$

where *I*_SERS_ represents the peak intensity of the SERS signal obtained from the hybrid Ag-Cu SERS substrate, and *C*_SERS_ corresponds to the concentration of the R6G solution deposited on the hybrid Ag-Cu SERS substrate; *I*_REF_ represents the peak intensity of the SERS signal obtained from the reference glass substrate, while *C*_REF_ corresponds to the concentration of the R6G solution deposited on the reference glass substrate. Figure 4 displays the measured SERS spectra for 10^−6^ M R6G on the hybrid Ag-Cu substrates with various Cu morphologies, along with 10^−2^ M R6G on the glass substrate for comparison. The peak intensity observed at 1357 cm^−1^, corresponding to the C–C stretching mode of R6G, is selected for the calculation of the *AEF*. Based on the given values, with *I*_SERS_ being 1895 for 10^−6^ M R6G on the hybrid Ag-Cu substrate, and *I*_REF_ being 168 for 10^−2^ M R6G deposited on the reference glass substrate, the *AEF* is calculated to be 1.13 × 10^5^. This significant *AEF* value indicates a high-quality SERS performance on the hybrid Ag-Cu SERS substrate. The substantial enhancement provided by the hybrid Ag-Cu SERS substrate further validates its efficacy for sensitive and effective trace molecule detection and analysis. In comparison, the Cu foil-based substrate *AEF* is 3.07 × 10^4^. This confirms the significant enhancement of the hybrid Ag-Cu SERS substrate through our structural design. Through a strategic design of the Cu structure used in the galvanic displacement reaction, it becomes possible to further optimize the performance of the SERS substrate. Our innovative approach introduces a novel idea aimed at significantly enhancing the capabilities of SERS substrates.

### 3.2. Analysis Sensitivity and Repeatability

**Figure 5:**
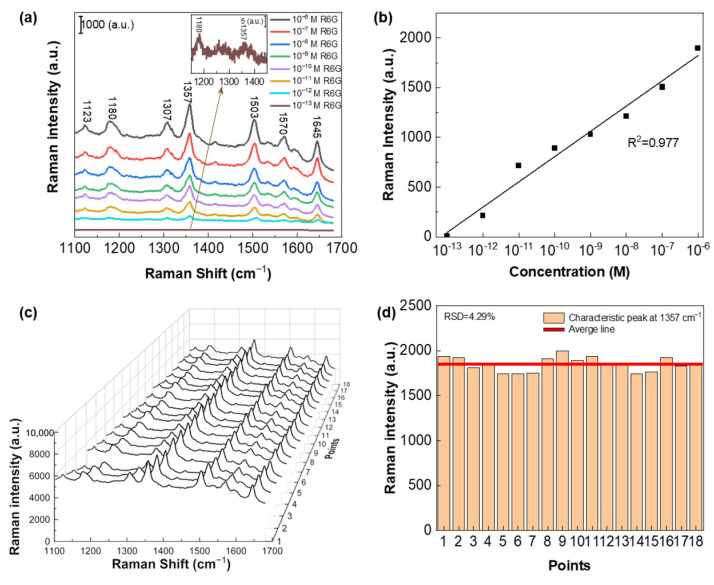
(**a**) SERS spectra for R6G obtained with different concentrations ranging from 10^−6^ to 10^−13^ M on the hybrid Ag-Cu substrate. The inset presents the Raman peaks of R6G at 1180 cm^−1^ and 1357 cm^−1^, corresponding to a concentration of 10^−13^ M. (**b**) Plot of the Raman intensity of R6G at 1357 cm^−1^ as a function of the logarithmic concentration. (**c**) Measured SERS spectra of R6G with a concentration of 10^−6^ M acquired from 18 randomly selected positions on the hybrid Ag-Cu substrate. (**d**) Intensity distribution of the 18 randomly collected spectra at 1357 cm^−1^ peak, illustrating the relative standard deviation (RSD) of 4.29%.

Using the optimal hybrid Ag-Cu SERS substrates, we conducted further investigations to explore their sensitivity. We tested two molecules with different molecular weights, R6G (molecular weight: 497.01) and BPy (molecular weight: 156.18). The choice of two molecules with a significant difference in molecular weight aims to verify the versatility and broad applicability of the SERS substrates. By testing molecules with substantial molecular weight differences, we can demonstrate the substrate’s ability to effectively detect and characterize a wide range of analytes, highlighting its utility and potential for various scientific and analytical applications. Figure 5a presents the measured SERS spectra obtained with different R6G concentrations, demonstrating that the limit of detection (LOD) for the R6G probe molecule reaches as low as 10^−13^ M. Figure 5b illustrates the Raman intensity of R6G at 1357 cm^−1^ plotted as a function of logarithmic concentration, showcasing a remarkable linear dependence with a high correlation coefficient (R^2^) of 0.977. Indeed, apart from exceptional detection sensitivity, the repeatability of the SERS substrate is a crucial factor for its practical application. The ability to achieve a reliable and consistent performance is essential to ensure accurate and reproducible results during repeated measurements. The capability of the SERS substrate to maintain its sensitivity and performance consistently over multiple measurements holds paramount importance for various practical applications, including analytical chemistry, bioanalysis, and environmental monitoring. It instills confidence in the reliability and robustness of the substrate, rendering it a valuable tool for precise and dependable molecule detection and analysis. The ability of the hybrid Ag-Cu SERS substrate to maintain its sensitivity and performance consistently over multiple measurements is of significant importance for various practical applications. For assessment purposes, we collected SERS spectra from 18 randomly selected positions on the substrate. Figure 5c displays the obtained SERS spectra of 10^−6^ M R6G molecules at these 18 different positions on the same substrate. This analysis allows us to evaluate the repeatability and consistency of the hybrid Ag-Cu SERS substrate across multiple spatial locations. The resulting spectral profiles exhibit a high degree of consistency. Notably, there is no significant shift observed in the characteristic Raman peaks, and there is no evident change in peak intensity across the 18 different positions on the substrate. These observations confirm the excellent uniformity and repeatability of the hybrid Ag-Cu SERS substrate. The substrate’s ability to consistently provide consistent and reliable SERS signals from various spatial locations underscores its suitability for practical use in sensitive and precise trace molecule detection and analysis. In order to better present the hybrid Ag-Cu SERS substrate performance, 18 different spectra were further quantitatively analyzed. Figure 5d depicts the intensity distribution of the 18 randomly collected spectra at the 1357 cm^−1^ peak. The red line represents the average value of the Raman signal intensity at 1357 cm^−1^ for 18 different spectra. The relative standard deviation (RSD) calculated from these spectra is found to be 4.29%. This remarkably low RSD value serves as strong evidence of the outstanding reproducibility exhibited by the hybrid Ag-Cu SERS substrate. The hybrid Ag-Cu SERS substrate’s ability to consistently deliver precise and consistent SERS signals across multiple measurements further underscores its exceptional performance and practical utility in various scientific and analytical applications.

**Figure 6:**
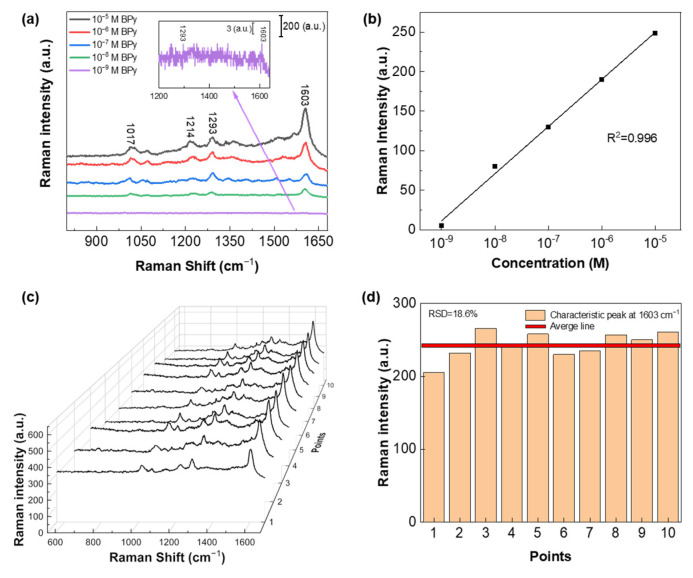
(**a**) SERS spectra for BPy obtained with different concentrations ranging from 10^−5^ to 10^−9^ M on the hybrid Ag-Cu substrate. The inset presents the Raman peaks of BPy at 1603 cm^−1^, corresponding to a concentration of 10^−9^ M. (**b**) Plot of the Raman intensity of BPy at 1603 cm^−1^ as a function of the logarithmic concentration. (**c**) Measured SERS spectra of BPy with a concentration of 10^−5^ M acquired from 10 randomly selected positions on the hybrid Ag-Cu substrate. (**d**) Intensity distribution of the 10 randomly collected spectra at 1603 cm^−1^ peak, illustrating a RSD of 18.6%.

Figure 6a illustrates the measured SERS spectra of different concentrations of BPy on the prepared hybrid Ag-Cu substrate. Notably, the LOD for BPy at 1603 cm^−1^ is low, reaching 10^−9^ M. The hybrid Ag-Cu SERS substrate showed high detection sensitivity for two different molecular weight molecules. In Figure 6b, the Raman intensity of BPy at 1603 cm^−1^ is plotted as a function of the logarithmic concentration, displaying excellent linear dependence with a high correlation coefficient (R^2^) of 0.996. To further verify the repeatability of the substrate, we conducted examinations using the BPy molecule as well. This additional investigation allows us to assess the substrate’s consistency and reliability in detecting and characterizing different types of molecules. Figure 6c presents the observed SERS spectra of 10^−5^ M BPy molecules at 10 different positions on the same hybrid Ag-Cu SERS substrate, demonstrating similar high-performance characteristics as observed for the R6G molecule. Similarly, the Raman spectral data for BPy underwent further quantitative analysis. The quantification of the data offers valuable insights into the substrate’s sensitivity and reliability in detecting and analyzing BPy. In Figure 6d, the intensity distribution of the 10 randomly collected spectra at the 1603 cm^−1^ peak is depicted, exhibiting a Relative Standard Deviation (RSD) of 18.6%. The red line represents the average value of the Raman signal intensity at 1603 cm^−1^ for 10 different spectra. This further confirms the exceptional sensitivity and reproducibility of our hybrid Ag-Cu SERS substrates for both R6G and BPy molecules. The hybrid Ag-Cu SERS substrates showcase superior performance, making them highly valuable for various analytical applications demanding high sensitivity and reliability. The hybrid Ag-Cu SERS substrate exhibited an exceptional detection performance for molecules with different molecular weights, showcasing its wide-ranging spectrum of SERS enhancement capabilities. This implies that the substrate offers significant enhancement for the detection of various molecules, making it a remarkably versatile and adaptable detection platform. We have performed a stability check of the proposed substate over time, and the existing data are displayed in Figure S4 in the Supplementary Materials. Despite the unavoidable Ag and Cu oxidation, it is notable from Figure S4 that the substrate still maintains a robust SERS signal enhancement over 30 days.

## 4. Conclusions

In summary, this work showcases a straightforward and cost-effective approach to attain highly reproducible and sensitive hybrid Ag-Cu SERS substrates. The controllable etching time enables the tuning of Cu nanoarrays to optimize the enhancement of incident light absorption. Furthermore, the Raman signal is significantly amplified by optimizing the growth time of Ag nanoparticles on the substrate. These findings underscore the importance of precise parameter control in achieving outstanding SERS performance. The AEF of the substrate under 532 nm laser excitation is 1.13 × 10^5^. The achieved LOD for the R6G and BPy molecules are 10^−13^ M and 10^−9^ M, respectively, exhibiting high sensitivity to molecules of different molecular weights. Our proposed technique presents a remarkable achievement, as it enables the production of highly sensitive and reproducible SERS substrates with low-cost and large-area fabrication capabilities. This promising outcome holds significant potential for practical applications in SERS-based rapid detection, particularly in the fields of environmental monitoring and food quality management. The ability to reliably and efficiently detect trace molecules in a cost-effective and scalable manner opens up new avenues for enhancing the speed and accuracy of environmental and food safety assessments.

## Data Availability

The data presented in this study are available on request from the corresponding author.

## Supplementary Materials

The following supporting information can be downloaded at: https://www.mdpi.com/article/10.3390/bios13080825/s1, Figure S1: (a) Surface-enhanced Raman spectroscopy (SERS) spectra for the 10^−6^ M R6G on Cu foils with the Ag nanoparticle growth reaction time of 2, 4, 6, 7, 8, 9, 10, 12 and 14 min, respectively. (b) Raman intensity of R6G at 1357 cm^−1^ and 1645 cm^−1^ as a function of the reaction time; Figure S2: (a–f) The Ag nanoparticle size distributions of hybrid Ag-Cu SERS substrates with the reaction time of 1, 3, 5, 7, 9 and 11 min, respectively; Figure S3: (a–f) The Ag nanoparticle distance distributions of hybrid Ag-Cu SERS substrates with the reaction time of 1, 3, 5, 7, 9 and 11 min, respectively; Figure S4: SERS spectra for the 10^−6^ M R6G on the hybrid Ag-Cu substrate by exposing it for 0, 15 and 30 days.
