# Supplementary material for: Nanosphere Lithography-Enabled Hybrid Ag-Cu Surface-Enhanced Raman Spectroscopy Substrates with Enhanced Absorption of Excitation Light
Source: Biosensors (Basel). 2023 Aug 17;13(8):825. doi: 10.3390/bios13080825 (PMC10452600; doi:10.3390/bios13080825)
Supplement: Supplementary file 1 [file biosensors-13-00825-s001.zip › biosensors-2556598-supplementary.pdf]

biosensors

Supplementary Material

Nanosphere Lithography-Enabled Hybrid Ag-Cu Surface-Enhanced Raman Spectroscopy Substrates with Enhanced Absorption of Excitation Light

Zixuan Wu, Jianxun Liu*, Zhenming Wang, Lei Chen, Yiwei Xu, Zongjun Ma, Delai Kong, Dan Luo and Yan Jun Liu *

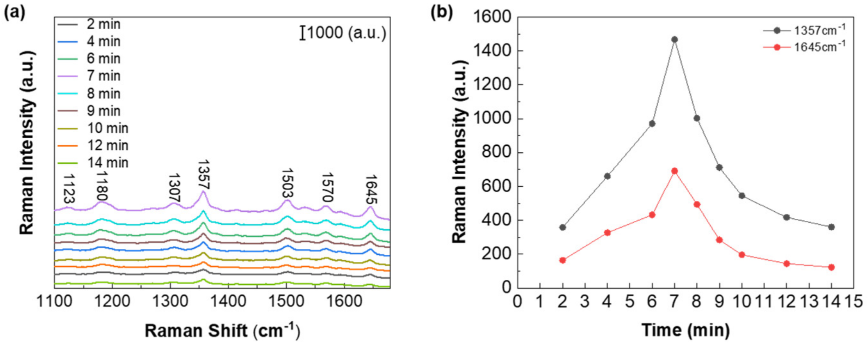

Figure S1. (a) Surface-enhanced Raman spectroscopy (SERS) spectra for the 10^{-6} M R6G on Cu foils with the Ag nanoparticle growth reaction time of 2, 4, 6, 7, 8, 9, 10, 12, and 14 min, respectively. (b) Raman intensity of R6G at 1357 cm^{-1} and 1645 cm^{-1} as a function of the reaction time.

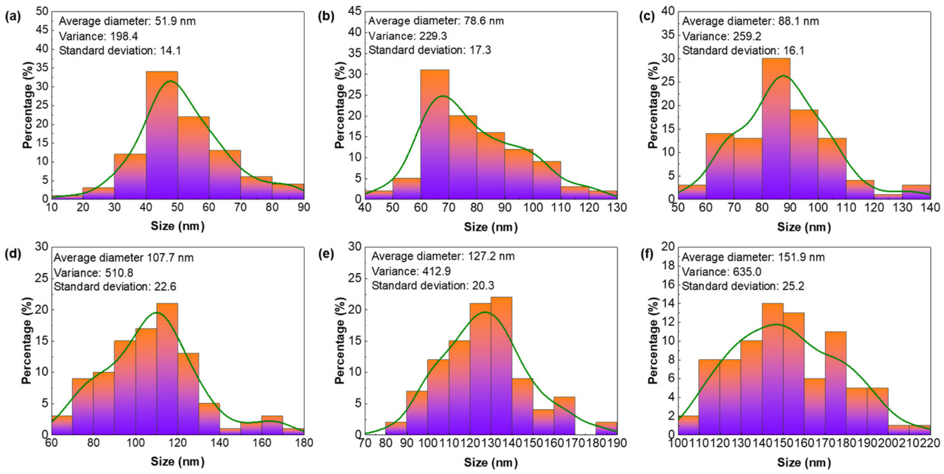

Figure S2. (a-f) The Ag nanoparticle size distributions of hybrid Ag-Cu SERS substrates with the reaction time of 1, 3, 5, 7, 9, and 11 min, respectively.

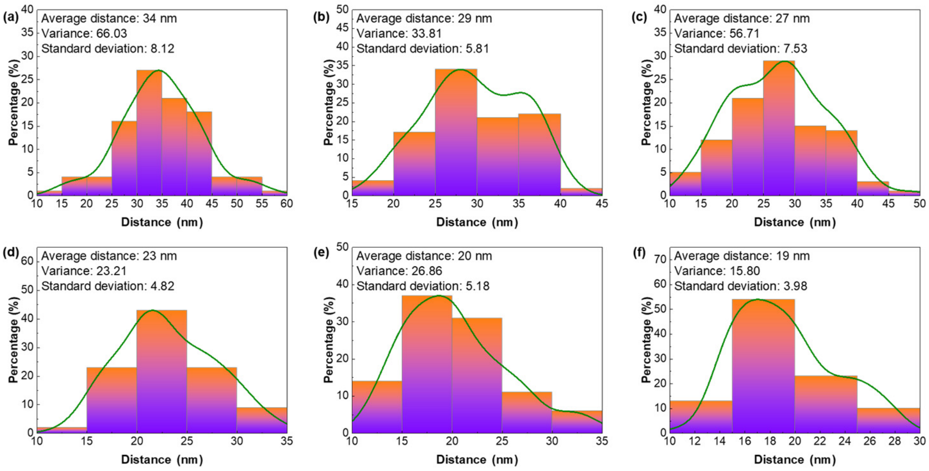

Figure S3. (a-f) The Ag nanoparticle distance distributions of hybrid Ag-Cu SERS substrates with the reaction time of 1, 3, 5, 7, 9, and 11 min, respectively.

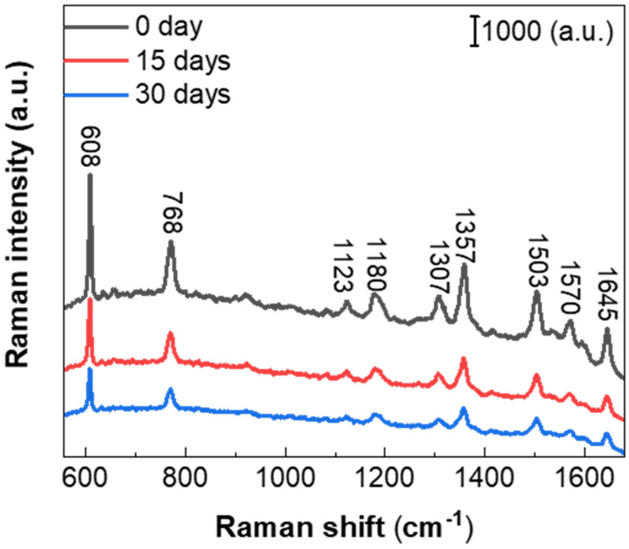

Figure S4. SERS spectra for the 10^{-6} M R6G on the hybrid Ag-Cu substrate by exposing it for 0, 15, and 30 days.
